# A Case-Based Workshop to Enhance Leadership Self-Efficacy Among Women in Academic Emergency Medicine

**DOI:** 10.5811/westjem.64686

**Published:** 2026-11-02

**Authors:** Tehreem Rehman, Anna P. Fang, Laura E. Walker, Sarah K. Wendel, Kelly Bookman, Amy Zeidan, Lauren Walter, Lauren M. Nentwich, Marie-Carmelle Elie-Turenne, Meagan R. Hunt

**Affiliations:** * Icahn School of Medicine at Mount Sinai, Department of Emergency Medicine, New York, New York; † Mass General Brigham, Department of Emergency Medicine, Boston, Massachusetts; ‡ Mayo Clinic, Department of Emergency Medicine, Rochester, Minnesota; § University of Virginia, Department of Emergency Medicine, Charlottesville, Virginia; ¶ The University of Alabama at Birmingham, Heersink School of Medicine, Department of Emergency Medicine, Birmingham, Alabama; || University of Colorado School of Medicine, Department of Emergency Medicine, Aurora, Colorado; # Emory University School of Medicine, Department of Emergency Medicine, Atlanta, Georgia; ** The University of Alabama at Birmingham, Department of Anesthesiology and Perioperative Medicine, Division of Critical Care Medicine, Birmingham, Alabama; †† The University of Alabama at Birmingham, Department of Medicine, Division of Palliative Medicine, Birmingham, Alabama; ‡‡ Wake Forest University School of Medicine, Department of Emergency Medicine, Winston-Salem, North Carolina

## Abstract

**Introduction:**

Women remain underrepresented in emergency medicine leadership roles, in part due to sex-based stereotypes that undervalue leadership traits traditionally associated with women, such as collaboration and empathy. Agentic traits like assertiveness are often perceived as requisite for leadership, while communal skills are overlooked. Leadership self-efficacy, an individual’s belief in their ability to successfully perform leadership roles, is a key predictor of leadership emergence.

**Methods:**

Our team developed a 90-minute didactic session featuring a panel of diverse women leaders followed by an interactive case-based exercise, delivered at the Society for Academic Emergency Medicine 35^th^ Annual Meeting in 2024. The intervention was evaluated using a pre- and postsurvey based on an adapted Leadership Self-Efficacy Scale measuring six domains of leadership self-efficacy. Eighteen participants completed both the pre- and postsession surveys, providing 18 matched pre–post pairs for analysis. Participants rated their confidence on a seven-point Likert scale. Our outcome of interest was the change in mean self-efficacy score for each domain on this seven-point scale, assessed with paired *t*-tests.

**Results:**

All six leadership domains showed increased mean scores postintervention. Dimension 1 (Leading Change) increased significantly (mean change +0.72/7, *P* = .001), as did Dimension 2 (Choosing team/Delegating) (mean change +0.61/7, *P* = .004). Dimensions 3–6 showed positive but nonsignificant trends.

**Conclusion:**

This single-session intervention was associated with increased leadership self-efficacy, particularly in the domains of leading change and team-building. The combination of role model exposure and interactive case practice may be a useful approach for empowering women physicians. This innovation can be adapted for implementation at other academic institutions.

## INTRODUCTION

Women remain persistently underrepresented in emergency medicine (EM) leadership: In academic EM, women represent only 15.0% of department chairs (20 of 133) despite comprising 38.8% of academic EM faculty (2,845 of 7,330).^1^ Sex-based stereotypes often create a double bind for women: When they exhibit agentic behaviors (assertive, competitive, and achievement-oriented behaviors traditionally associated with men), they may face backlash for violating sex-based expectations, yet communal behaviors (collaborative, empathetic, and relationship-oriented) are often overlooked or undervalued in leadership contexts.^2^,^3^ These dynamics may undermine women’s leadership self-efficacy by limiting visible role models and discouraging pursuit of leadership opportunities.^4^ Few interventions in academic EM are explicitly designed to counter sex-based leadership stereotypes. Women-only leadership development programs suggest such interventions can facilitate leadership role identity construction through validation and belonging.^5^

Leadership self-efficacy, defined as an individual’s belief in her ability to successfully fulfill leadership roles, is a key predictor of leadership emergence and effectiveness.^6^ Self-efficacy beliefs influence motivation, goal-setting, and persistence,^7^,^8^ and higher self-efficacy is associated with greater willingness to assume leadership roles.^9^ Women often report lower leadership self-efficacy than men, particularly in domains involving assertiveness and change initiation,^4^,^10^ a gap partially explained by fear of backlash, limited role models, and internalized societal messages about leadership.^2^,^11^ Interventions that provide role model exposure, skill validation, and opportunities for practice have shown promise.^12^ Consistent with social cognitive theory, which identifies vicarious learning and mastery experiences as primary sources of self-efficacy,^8^ the intervention paired role model exposure with case-based practice.

### Objectives

The Academy for Diversity and Inclusion in Emergency Medicine Operations Committee and the Academy for Women in Academic Emergency Medicine collaboratively designed a didactic intervention to counter sex-based leadership stereotypes, elevate undervalued communal traits, and strengthen participants’ internal validation of their leadership abilities. Our objective was to evaluate whether this single 90-minute workshop measurably improved leadership self-efficacy among women in academic EM.

## METHODS

### Curricular Design

The intervention was based on the premise that effective leadership requires both consideration (employee-oriented, communal) and structuring (task-oriented, agentic) behaviors, yet consideration behaviors are often perceived as feminine and undervalued.^13^ The design paired role model exposure with interactive skill practice.

The 90-minute didactic session, implemented at the Society for Academic Emergency Medicine Annual Meeting in 2024, was open to all registered attendees and specifically promoted to women in academic EM. After an introduction and administration of the preintervention survey (5 minutes), a moderated panel of three senior women leaders shared how traditionally undervalued traits, such as collaboration, empathy, and relationship-building, contributed to their leadership success, followed by a brief question-and-answer session (30 minutes). Each panelist came from a different academic institution and held a different type of senior leadership role, spanning departmental leadership (chair or vice chair) and national specialty society leadership. Their areas of focus included informatics, operations, quality, and research, so that participants encountered more than one route to advancement and more than one leadership style.

Participants then worked in facilitated small groups (five to eight participants) through an interactive case exercise (40 minutes) that required active participation from every group member, assuming the role of a newly appointed emergency department director tasked with improving length of stay by addressing radiology turnaround times. Discussion questions addressed stakeholder resistance, team selection, conflict management, building buy-in, and measuring success. The full case and facilitator guide are provided as eAppendix B. The session closed with administration of the postintervention survey (10 minutes) and a summary of key takeaways (5 minutes). Each group was facilitated by a leadership team member or experienced volunteer using a standardized guide.

Outcomes were measured with a pre- and postintervention survey based on the Leadership Self-Efficacy Scale, a validated multidimensional instrument developed by Bobbio and Manganelli that measures six core domains: (1) starting and leading change processes in groups; (2) choosing effective followers and delegating responsibilities; (3) building and managing interpersonal relationships; (4) showing self-awareness and self-confidence; (5) motivating people; and (6) gaining consensus of group members.^14^ Domains 1 and 2 align most closely with agentic traits, whereas Domains 3, 5, and 6 reflect communal, relationship-oriented traits. For feasibility in a conference setting, one representative item was selected per domain (eAppendix A), scored on a seven-point Likert scale from “strongly disagree” (1) to “strongly agree” (7).

Surveys were administered electronically through REDCap (Research Electronic Data Capture). Participants completed the surveys on their personal devices via a survey link at the beginning and end of the session, and responses were exported from REDCap for analysis. No demographic data were collected to protect anonymity given the small number of attendees. The outcome of interest was the change in mean self-efficacy score for each domain from pre- to immediately postintervention, assessed with paired *t*-tests. The institutional review board (IRB) determined that this evaluation did not require IRB review (45 CFR 46.102). Participants provided informed consent and used randomly generated codes allowing anonymous pre-post pairing. Total session attendance was not formally recorded; session organizers estimated approximately 40 attendees.

## RESULTS

### Impact/Effectiveness

A total of 51 survey submissions were collected. Based on randomly generated participant codes, 18 participants completed both pre- and postsurveys, forming matched pairs for analysis; the first chronological REDCap entry for each code was considered the pretest and the second the posttest. Total attendance was not formally recorded, but session organizers estimated approximately 40 attendees; thus, the 18 matched pairs represent an estimated 45% of those present.

Pre- and postintervention mean scores for all six dimensions are reported in Table. All six dimensions showed increased mean scores from pre- to postintervention. Dimension 1 (Leading Change) increased from 4.83/7 to 5.56/7 (mean change +0.72/7, *P* = .001), and Dimension 2 (Choosing Team/Delegating) improved from 5.11/7 to 5.72/7 (mean change +0.61/7, *P* = .004). Changes in Dimensions 3–6 were positive but did not reach statistical significance.

## DISCUSSION

### Lessons Learned

Improvements were concentrated in Dimensions 1 and 2, the more agentic domains, in which internalized stereotypes may most undermine women’s confidence.^15^,^16^ These were also the domains most directly targeted by the case exercise, which emphasized change management and team selection. High baseline scores in several domains, particularly Dimension 4 (Self-Confidence, baseline mean 5.78/7), suggest possible ceiling effects; however, ceiling effects alone cannot explain the nonsignificant findings, because the baseline mean for Dimension 5 (Motivating Others, 5.11/7) was identical to that of Dimension 2, which did improve significantly. The alignment between the session’s content emphasis and the domains that improved may therefore also have contributed to this pattern. Several design features may have contributed to these gains: The panel of senior women leaders provided vicarious learning and validation of leadership identity; the case exercise created mastery experiences; and the session explicitly validated communal leadership traits. These mechanisms are consistent with social cognitive theory^8^ and with prior literature on women’s leadership development programs, leadership self-efficacy interventions, and professional identity formation.^5^,^12^,^17^ However, because the intervention was evaluated as a whole, this evaluation cannot determine which components drove the observed improvements.

## LIMITATIONS

This evaluation has several limitations. First, the sample size (*N* = 18 matched pairs) is relatively small, limiting statistical power to detect changes in domains with high baseline scores. Second, because the total workshop attendance was not formally recorded, the response rate is based on an organizer estimate rather than a count, and the possibility of response bias cannot be excluded. Third, this was a single-session intervention at a national conference; longer-term follow-up is needed to assess sustainability. Fourth, participants were self-selected and motivated conference attendees, which may limit generalizability to other contexts. Fifth, the evaluation relied on self-reported measures rather than objective measures of leadership behavior, and the study design did not permit assessment of which individual program components were effective. Lastly, because no demographic data were collected by design, respondents could not be characterized, we could not test whether completers differed from noncompleters, and the sex of participants in this open session was not documented. We use the term “sex-based” throughout per journal convention while recognizing that this dimension was not measured.

### Exportability and Next Steps

This innovation requires minimal resources and can be delivered in a 90-minute session. Key export elements include recruiting diverse women leaders, adapting the case locally, training facilitators, and ensuring a supportive environment. The model can be adapted for department retreats, faculty development programs, residency curricula, or other groups underrepresented in leadership, and it is best integrated into ongoing programming rather than being delivered once. Because structural barriers remain, future work should couple individual-level development with institutional reforms and include longer-term follow-up, objective measures of leadership behavior, and assessment of whether increased self-efficacy translates into pursuit and attainment of leadership roles.

## CONCLUSION

This innovation offers a practical, low-resource approach to leadership development for women in academic EM. By validating diverse leadership styles and providing opportunities for skill practice, such interventions may empower women physicians to recognize and leverage their leadership strengths in specific domains. As a single-session intervention evaluated in a small sample, it is a promising starting point rather than a solution to sex-based disparities in academic EM leadership, and it is best paired with sustained programming and institutional change.

## Supplementary Information





## Figures and Tables

**Table:** Pre- and postintervention leadership self-efficacy scores in a study of women in academic emergency medicine attending a Society for Academic Emergency Medicine 2024 case-based leadership workshop.

Leadership Domain	Pretest Mean	Posttest Mean	Mean Change	*P* value
Leading Change	4.83	5.56	+0.72	**.001**
Choosing Team	5.11	5.72	+0.61	**.004**
Relationships	5.44	5.94	+0.50	.07
Self-Confidence	5.78	6.06	+0.28	.17
Motivating Others	5.11	5.67	+0.56	.09
Consensus	5.28	5.39	+0.11	.79

Paired *t*-tests assess change in mean scores. Bold indicates *P* < .05.
