# Supplementary material for: A Case-Based Workshop to Enhance Leadership Self-Efficacy Among Women in Academic Emergency Medicine
Source: West J Emerg Med. 2026 Nov 2;27(6):1548–51. doi: 10.5811/westjem.64686 (PMC13761715; doi:10.5811/westjem.64686)
Supplement: Supplementary file 1 [file wjem-27-1548-s001.docx]

**Supplemental Appendix A**

**LSES Domains and Representative Survey Items**

The Leadership Self-Efficacy Scale (LSES) measures six core domains of leadership self-efficacy. For feasibility in a conference setting, one representative item was selected for each domain, creating a shortened six-question survey. Items were scored on a seven-point Likert scale from Strongly Disagree (1) to Strongly Agree (7).

| **Dimension** | **LSES Domain** | **Representative Survey Item** |
| --- | --- | --- |
| 1 | Starting and leading change processes in groups | *“I am able to change things in a group, even if they are not completely under my control”* |
| 2 | Choosing effective followers and delegating responsibilities | *“I am confident in my ability to choose group members to build an effective team”* |
| 3 | Building and managing interpersonal relationships within the group | *“I can successfully manage relationships with all group members”* |
| 4 | Showing self-awareness and self-confidence | *“I am confident in my ability to get things done”* |
| 5 | Motivating people | *“I am able to motivate group members and give them opportunities to grow”* |
| 6 | Gaining consensus of group members | *“I can usually lead a group with the consensus of all members”* |

*Adapted from: Bobbio A, Manganelli AM. Leadership self-efficacy scale: a new multidimensional instrument. TPM 2009;16:3-24.*
