# Supplementary material for: A Case-Based Workshop to Enhance Leadership Self-Efficacy Among Women in Academic Emergency Medicine
Source: West J Emerg Med. 2026 Nov 2;27(6):1548–51. doi: 10.5811/westjem.64686 (PMC13761715; doi:10.5811/westjem.64686)
Supplement: Supplementary file 2 [file wjem-27-1548-s002.docx]

**Supplemental Appendix B. Case-Based Exercise and Facilitator Guide**

Format: Participants work in facilitated small groups of 5 to 8 for approximately 40 minutes, and every participant is asked to contribute. Each group is led by a facilitator using this guide. The case unfolds in five segments; the facilitator introduces each segment after the group has worked through the preceding questions. Italicized text is a prompt for the facilitator.

**THE CASE:**

You are the newly appointed director for an academic emergency department. You have been tasked with improving length of stay for ED discharged patients. After discussing with ED providers and staff, you have been told that long turnaround times in radiology frequently cause delays in patient care and disposition. CT scans are taking a long time to obtain, and EM providers are unsure how long CT scans will take to complete after they are ordered. The hospital leadership would like you to address this problem.

**DISCUSSION QUESTIONS:**

1. You discuss with radiology leadership (including their chair, who is new, and their vice chair of operations). They disagree that imaging is taking a long time and feel that this is not an issue. How do you respond?

***Facilitator: consider asking a panelist whether they have had a similar experience in their leadership positions.***

2. You can’t solve this problem on your own. How do you identify a team? What characteristics do you look for in individuals who will be change makers?
3. What is critical to consider when addressing barriers and looking for solutions in a case like this? For example, including diverse perspectives, obtaining metrics, or organizational mission, values, and culture.

***Facilitator: the goal is to use this question to let participants highlight specific aspects of the approach that reflect their own leadership traits and talents.***

**Great work getting this effort off the ground! Here’s where things get exciting.**

You are in your process mapping meeting and conflict arises between transport staff and the ED RNs regarding the length of time to transport arrival after patients are marked as ready for CT. Members of the committee are pointing fingers at each other.

**DISCUSSION QUESTION:** How do you navigate this?

**Nice job managing stakeholder relationships and communication. What’s your next challenge?**

You build an interdisciplinary team that comes up with a solution that shows incredible results, but it only seems to be functional during parts of the day when you or one of your process champions are working.

**DISCUSSION QUESTION:** How do you explain to the rest of the department why this change is needed and why they should support it?

**Way to build buy-in from the department! Your hospital leadership wants data showing that the problem has been addressed.**

The problem was that long turnaround times in radiology frequently cause delays in patient care and disposition. CT scans are taking a long time to obtain, and EM providers are unsure how long CT scans will take to complete after they are ordered.

**DISCUSSION QUESTIONS:** How would you measure success, either as an interim measure or overall? How would you determine that your implementation plan is working?

***Facilitator: the goal is to surface diverse measures of success.***

**Go forth and lead. Emergency Medicine needs leaders like you! You don’t have to hold a position of leadership to start leading.**

**Discussion:** Please share one personal trait that is a strength in your current leadership position OR that will be a strength in any future leadership positions (if not currently holding a formal leadership position).

***Facilitator: if time allows, ask participants to cite an example of when they have used their strengths as part of a problem-solving process or as a leader.***
